# Use of Prediction Intervals in Network Meta-analysis

**DOI:** 10.1001/jamanetworkopen.2019.9735

**Published:** 2019-08-21

**Authors:** Lifeng Lin

**Affiliations:** 1 Department of Statistics, Florida State University, Tallahassee

## Abstract

This cross-sectional study examines the prevalence of reporting prediction intervals in network meta-analysis articles and provides a worked example.

## Introduction

Network meta-analysis (NMA) is an increasingly popular tool to synthesize direct and indirect evidence for simultaneously comparing multiple treatments. Although guidelines have been developed to improve NMAs,^1^ the quality of methodology and reporting of many NMAs is inconsistent with the rapid growth of their publication rates.^2^ Within the context of conventional pairwise meta-analyses, it has been advocated to routinely report 95% prediction (or predictive) intervals (PIs) alongside 95% CIs.^3,4^ Prediction intervals give the range within which the results of a future study might lie. They can also be used to effectively appraise heterogeneity and avoid the problems of the popular *I*^2^ statistic.^5^ However, the practice of using PIs is uncommon in NMAs.

As in conventional meta-analyses, heterogeneity frequently exists in NMAs owing to differences in study setting, participants, treatment definitions, and so on. This may affect the validity and interpretation of the NMA. To account for heterogeneity, random-effects models are usually applied, and evidence users often focus on the summarized overall results with 95% CIs.

Confidence intervals and PIs are used for different purposes. Suppose an NMA compares multiple treatments for obesity, with its primary outcome as weight loss. Confidence intervals describe uncertainty in overall weight loss for various treatment comparisons among the populations in the NMA. As such, their widths typically shrink toward 0 as the number of studies increases. In addition to uncertainty in overall results, PIs’ widths incorporate the variation caused by heterogeneity between different studies. When a new patient comes to the clinic, PIs (rather than CIs) should be used to predict the new patient’s weight loss and thus recommend the optimal treatment.

## Methods

To investigate the use of PIs in NMAs, relevant articles published in *JAMA*, *The Lancet*, and *BMJ* from January 1, 2010, to December 31, 2018, were identified. Full-length articles with original data were the focus. The search procedures are provided in eAppendix 1 in the Supplement. This study did not require institutional review board approval because it focuses on statistical methods and uses published data in the literature. Its reporting follows the Strengthening the Reporting of Observational Studies in Epidemiology (STROBE) reporting guideline.

In addition, to help clinicians understand how PIs can be produced in NMAs, available software programs and their implementations were illustrated via a worked example. This example used a classic NMA data set initially reported by Hasselblad^6^ and based on 24 studies. This NMA compared 4 treatments for smoking cessation (a binary outcome) as follows: (1) no intervention, (2) self-help, (3) individual counseling, and (4) group counseling.^6^ The estimated overall odds ratios of all treatment comparisons with 95% CIs and 95% PIs are reported. Statistical significance was prespecified at α = .05.

## Results

Fifty-eight full-length articles on NMAs with original data were identified (eTable 1 in the Supplement). *JAMA*, *The Lancet*, and *BMJ* published 12 (21%), 11 (19%), and 35 (60%) NMA articles, respectively. Only 5 articles (9%) reported PIs, all published in *BMJ*.

Software programs for producing PIs in NMAs appear in eTable 2 in the Supplement. Detailed instructions and results using these software programs appear in eAppendix 2 and the eFigure in the Supplement. The Figure shows the results of the NMA of smoking cessation using Stata version 13 (StataCorp). The 95% CIs of individual counseling vs no intervention and group counseling vs no intervention were above the null value 1.0. The overall treatment effects of individual counseling and group counseling were significantly better than no intervention among the 24 studies; they led to higher smoking cessation rates. The remaining 4 comparisons were nonsignificant. Nevertheless, the 95% PIs of all comparisons were wider than the corresponding 95% CIs and contained 1.0; thus, they indicated substantial heterogeneity between studies. Owing to such heterogeneity, the overall effects of individual counseling and group counseling could be null or even in the opposite direction for patients in a new study.^4^

**Figure.**
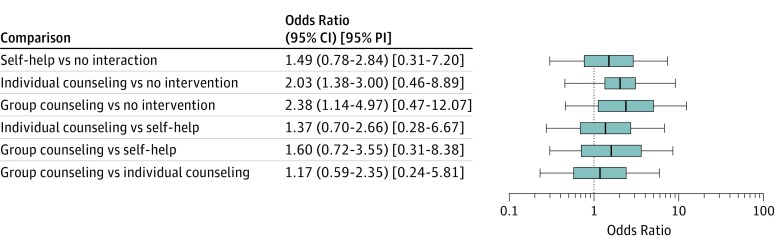
Estimated Overall Odds Ratios of 6 Treatment Comparisons in the Network Meta-analysis of Smoking Cessation Using Stata Centers of boxes indicate odds ratios, with edges representing 95% CIs. Whiskers represent 95% prediction intervals (PIs).

## Discussion

A limitation of this study is that detailed statistical models used in different software programs were not explored. They may make different assumptions and thus have some impact on the NMA results (eFigure in the Supplement).

In summary, the reporting rate of PIs was low among NMAs, but they are crucial for interpreting results within a future study setting and have been advocated for conventional meta-analyses. The low reporting rate was possibly because most NMA guidelines lack recommendations about PIs and performing NMAs is relatively complicated. Nevertheless, as shown in the worked example, several software programs are available to feasibly produce PIs in NMAs.

## Notes

Supplement. **eAppendix 1.** Literature Search for Articles on Network Meta-analysis **eAppendix 2.** Example of Producing Prediction Intervals in Network Meta-analysis **eTable 1.** Characteristics of Articles on Network Meta-analyses **eTable 2.** Summary of Software Programs for Producing Prediction Intervals in Network Meta-analysis **eFigure.** Estimated Overall Odds Ratios of 6 Treatment Comparisons in the Network Meta-analysis of Smoking Cessation Using Stata, R Package netmeta, and WinBUGS **eReferences.**
