# Supplementary material for: Use of Prediction Intervals in Network Meta-analysis
Source: JAMA Netw Open. 2019 Aug 21;2(8):e199735. doi: 10.1001/jamanetworkopen.2019.9735 (PMC6707007; doi:10.1001/jamanetworkopen.2019.9735)
Supplement: Supplement. — eAppendix 1. Literature Search for Articles on Network Meta-analysis eAppendix 2. Example of Producing Prediction Intervals in Network Meta-analysis eTable 1. Characteristics of Articles on Network Meta-analyses eTable 2. Summary of Software Programs for Producing Prediction Intervals in Network Meta-analysis eFigure. Estimated Overall Odds Ratios of 6 Treatment Comparisons in the Network Meta-analysis of Smoking Cessation Using Stata, R Package netmeta, and WinBUGS eReferences. [file jamanetwopen-2-e199735-s001.pdf]

Supplementary Online Content

Lin L. Use of prediction intervals in network meta-analysis. *JAMA Netw Open.* 2019;2(8): e199735. doi:10.1001/jamanetworkopen.2019.9735

eAppendix 1. Literature Search for Articles on Network Meta-analysis

eAppendix 2. Example of Producing Prediction Intervals in Network Meta-analysis

eTable 1. Characteristics of Articles on Network Meta-analyses

eTable 2. Summary of Software Programs for Producing Prediction Intervals in Network Meta-analysis

eFigure. Estimated Overall Odds Ratios of 6 Treatment Comparisons in the Network Meta-analysis of Smoking Cessation Using Stata, R Package netmeta, and WinBUGS

eReferences

This supplementary material has been provided by the authors to give readers additional information about their work.

© 2019 Lin L. *JAMA Network Open.*

eAppendix 1. Literature Search for Articles on Network Meta-analysis

We searched for full-length articles with original data of network meta-analyses from 2010 to 2018, and we excluded letters, commentaries, news, and research methodology and reporting. The literature search was conducted via the official websites of *JAMA* (https://jamanetwork.com/advanced-search), *the Lancet* (https://www.thelancet.com/search/advanced), and *BMJ* (https://www.bmj.com/search/advanced). We downloaded articles with the term “meta analysis”, “meta analyses”, “meta epidemiological”, “network meta analysis”, “network meta analyses”, “mixed treatment comparison(s)”, or “multiple treatment(s)” in their titles. Then, we looked at the articles’ contents, and excluded articles if they were not network meta-analyses of multiple treatments. Some articles were also excluded when they presented data of multiple treatments but did not formally perform network meta-analyses to simultaneously synthesize their evidence. Of note, although most articles with the term “meta analysis”, “meta analyses”, or “meta epidemiological” were conventional pairwise meta-analyses, some were actually network meta-analyses; therefore, these terms were retained in our literature search. In addition, we compared the obtained articles with the network meta-analyses collected by Nikolakopoulou et al.[1] (searched before 2014), Bafeta et al.[2,3] (searched before 2013), and Trinquart et al.[4] (searched before 2013 with restrictions on model types). Missed articles were added to our final list (eTable 1).

© 2019 Lin L. *JAMA Network Open.*

eAppendix 2. Example of Producing Prediction Intervals in Network Meta-analysis

eTable 2 presents software programs for NMAs and the corresponding commands for producing prediction intervals. All commands are simple and do not require much additional effort in the analyses. This appendix applies the available software programs to the example dataset of smoking cessation. The following statistical analyses estimated odds ratios on a logarithmic scale with 95% confidence/credible intervals and prediction intervals. The specific commands for producing prediction intervals are highlighted in the following statistical code.

Stata

If the Stata routines for network meta-analysis have not been installed yet, type the following command in Stata

```
. net from http://www.homepages.ucl.ac.uk/~rmjwiww/stata/
```

Click the link `meta` and install the packages `network` and `mvmeta`. Also, type the following command

```
. net from http://www.stata-journal.com/software/sj15-4/
```

Install the package `st0411`. The detailed instructions of these Stata routines have been provided by Chaimani et al.,[5] Chaimani and Salanti,[6] and White.[7]

The smoking cessation dataset is stored in the file “smokingcessation.dta”. By typing

```
. list, noobs clean
```

the dataset can be displayed as follows

```
study rA    nA    rB    nB    rC    nC    rD    nD
1     9     140   .     .     23    140   10    138
2     .     .     11    78    12    85    29    170
3     75    731   .     .     363   714   .     .
4     2     106   .     .     9     205   .     .
5     58    549   .     .     237   1561  .     .
6     .5    34    .     .     9.5   49    .     .
7     3     100   .     .     31    98    .     .
8     1     31    .     .     26    95    .     .
9     6     39    .     .     17    77    .     .
10    79    702   77    694   .     .     .     .
11    18    671   21    535   .     .     .     .
12    64    642   .     .     107   761   .     .
13    5     62    .     .     8     90    .     .
14    20    234   .     .     34    237   .     .
15    .5    21    .     .     .     .     9.5   21
16    8     116   19    149   .     .     .     .
17    95    1107  .     .     143   1031  .     .
18    15    187   .     .     36    504   .     .
19    78    584   .     .     73    675   .     .
20    69    1177  .     .     54    888   .     .
21    .     .     20    49    16    43    .     .
22    .     .     7     66    .     .     32    127
23    .     .     .     .     12    76    20    74
24    .     .     .     .     9     55    3     26
```

© 2019 Lin L. *JAMA Network Open.*

Note that the continuity correction of 0.5 is applied to each study that contains zero event in a certain treatment arm. Then, we run the following commands to implement network meta-analysis and derive 95% prediction intervals:

```
. network setup r n, studyvar(study)
. network table
. network meta consistency
. intervalplot, pred
```

The last command returns the following results:

```
The intervalplot command assumes that the saved results from mvmeta or
network meta commands have been derived from the current
> dataset
```

_Comparison	_Effect_Size	_Standard_Error	_LCI	_UCI	_LPrI	_UPrI
_y_B	.3971683	.3297603	-.24915	1.043487	-1.180223	1.97456
_y_C	.7090092	.1982125	.3205197	1.097499	-.7671354	2.185154
_y_D	.868733	.3749113	.1339203	1.603546	-.7529295	2.490396
_y_C-_y_B	.3118409	.3391682	-.3529166	.9765984	-1.274405	1.898087
_y_D-_y_B	.4715647	.4058903	-.3239657	1.267095	-1.182962	2.126092
_y_D-_y_C	.1597238	.3534939	-.5331115	.8525591	-1.440384	1.759832

R package "netmeta"

If the R package "netmeta" has not been installed yet, type the following command in R to install this package:

```
> install.packages("netmeta")
```

Load this package:

```
> library("netmeta")
```

This package contains the smoking cessation dataset, which can be used by typing:

```
> data(smokingcessation)
```

The following commands produce the estimated log odds ratios and their 95% confidence intervals and 95% prediction intervals:

```
> pair <- pairwise(list(treat1, treat2, treat3),
+   event = list(event1, event2, event3), n = list(n1, n2, n3),
+   data = smokingcessation, sm = "OR")
> out.netmeta <- netmeta(pair, prediction = TRUE)
> est.netmeta <- out.netmeta$TE.random
> CI.lower.netmeta <- out.netmeta$lower.random
> CI.upper.netmeta <- out.netmeta$upper.random
> PrI.lower.netmeta <- out.netmeta$lower.predict
> PrI.upper.netmeta <- out.netmeta$upper.predict
```

Here, the argument `prediction` in the R function `netmeta()` specifies whether prediction intervals are produced (`TRUE`) or not (`FALSE`). The results can be displayed as follows:

```
> est.netmeta
          A          B          C          D
A 0.0000000 -0.4162377 -0.7334060 -0.9022982
```

```
B 0.4162377  0.0000000 -0.3171683 -0.4860605
C 0.7334060  0.3171683  0.0000000 -0.1688922
D 0.9022982  0.4860605  0.1688922  0.0000000
> CI.lower.netmeta
            A          B          C          D
A  0.00000000 -1.1377048 -1.1624885 -1.7089929
B -0.30522934  0.0000000 -1.0601065 -1.3690309
C  0.30432357 -0.4257698  0.0000000 -0.9346123
D  0.09560348 -0.3969098 -0.5968279  0.0000000
> CI.upper.netmeta
         A         B          C           D
A 0.000000 0.3052293 -0.3043236 -0.09560348
B 1.137705 0.0000000  0.4257698  0.39690985
C 1.162488 1.0601065  0.0000000  0.59682791
D 1.708993 1.3690309  0.9346123  0.00000000
> PrI.lower.netmeta
           A         B         C         D
A  0.0000000 -2.193457 -2.401299 -2.720079
B -1.3609814  0.000000 -2.104264 -2.343108
C -0.9344867 -1.469927  0.000000 -1.966722
D -0.9154828 -1.370987 -1.628938  0.000000
> PrI.upper.netmeta
         A        B         C         D
A 0.000000 1.360981 0.9344867 0.9154828
B 2.193457 0.000000 1.4699273 1.3709866
C 2.401299 2.104264 0.0000000 1.6289377
D 2.720079 2.343108 1.9667221 0.0000000
```

The five objects `est.netmeta`, `CI.lower.netmeta`, `CI.upper.netmeta`, `PrI.lower.netmeta`, and `PrI.upper.netmeta` accordingly contain the point estimates of overall log odds ratios of all treatment comparisons, their 95% confidence intervals' lower and upper bounds, and their 95% prediction intervals' lower and upper bounds. Each object is a 4 x 4 matrix, and each matrix element corresponds to a comparison of a pair of treatments. Specifically, each treatment comparison result is interpreted as the treatment corresponding to its row vs. the treatment corresponding to its column.

WinBUGS (implemented via R package "R2WinBUGS")

If the software program WinBUGS has not been downloaded, it is available at https://www.mrc-bsu.cam.ac.uk/wp-content/uploads/2018/11/winbugs143_unrestricted.zip. After unzipping the downloaded file, the software program "WinBUGS14.exe" is located in the folder "WinBUGS14". If the R package "R2WinBUGS" has not been installed yet, type the following command in R to install this package:

```
> install.packages("R2WinBUGS")
```

This package implements BUGS models via the R platform. Load the R package "R2WinBUGS":

```
> library("R2WinBUGS")
```

The BUGS model for network meta-analysis can be specified in the following R function:

```
BayesianNMAModel <- function(){
  for(i in 1:NS){
    w[i,1] <- 0
    delta[i,t[i,1]] <- 0
```

```
    mu[i] ~ dnorm(0, 0.0001)  # vague priors for trial baselines
    for(k in 1:na[i]){
      r[i,k] ~ dbin(p[i,t[i,k]], n[i,k])  # binomial likelihood
      logit(p[i,t[i,k]]) <- mu[i] + delta[i,t[i,k]]  # model
    }
    for(k in 2:na[i]){
      # trial-specific LOR
      delta[i,t[i,k]] ~ dnorm(md[i,t[i,k]], taud[i,t[i,k]])
      md[i,t[i,k]] <- d[t[i,k]] - d[t[i,1]] + sw[i,k] # mean of LOR
      taud[i,t[i,k]] <- tau*2*(k - 1)/k  # precision of LOR
      # adjustment of multi-arm trials
      w[i,k] <- delta[i,t[i,k]] - d[t[i,k]] + d[t[i,1]]
      sw[i,k] <- sum(w[i,1:(k-1)])/(k - 1)
    }
  }

  d[1] <- 0
  for(k in 2:NT){
    d[k] ~ dnorm(0, 0.0001)
  }

  tau <- pow(sd, -2)
  sd ~ dunif(0, 2)  #  uniform prior for random effects standard deviation

  # pairwise ORs
  for(c in 1:(NT - 1)){
    for(k in (c + 1):NT){
      lor[c,k] <- d[k] - d[c]
      lor.new[c,k] ~ dnorm(lor[c,k], tau)  # predicted LOR
    }
  }
}
```

Alternatively, the BUGS model can be stored in a separate text file in the working directory. Note that the last line `lor.new[c,k] ~ dnorm(lor[c,k], tau)` is the additional part for producing prediction intervals,[8] and all remaining parts are the conventional code to perform Bayesian network meta-analysis.[9,10] After specifying the BUGS model, we type the following R code to prepare the smoking cessation dataset in the format of the above BUGS model:

```
> NS <- dim(smokingcessation)[1]
> NT <- 4
> na <- c(rep(3, 2), rep(2, NS - 2))
> r <- as.matrix(smokingcessation[,c("event1", "event2", "event3")])
> n <- as.matrix(smokingcessation[,c("n1", "n2", "n3")])
> treat <- smokingcessation[,c("treat1", "treat2", "treat3")]
> t <- matrix(NA, NS, 3)
> for(i in 1:NS){
+   for(j in 1:3){
+     if(treat[i,j] == "A") t[i,j] <- 1
+     if(treat[i,j] == "B") t[i,j] <- 2
+     if(treat[i,j] == "C") t[i,j] <- 3
+     if(treat[i,j] == "D") t[i,j] <- 4
+   }
+ }
```

Based on the above reformatted data, we specify the objects of data and the parameters' initial values (used to initialize the Markov chain Monte Carlo algorithm) in the BUGS model as follows:

```
> data <- list(NS = NS, NT = NT, na = na, r = r, n = n, t = t)
> inits <- list(
+   list(mu = rep(0, NS), d = rep(0, 4), sd = 1),
+   list(mu = rep(0.5, NS), d = rep(-0.5, 4), sd = 2),
+   list(mu = rep(-0.5, NS), d = rep(0.5, 4), sd = 0.5))
```

Consequently, the following code performs Bayesian network meta-analysis by invoking WinBUGS from R:

```
> set.seed(1234)
> out.bugs <- bugs(data = data, inits = inits,
+   parameters.to.save = c("lor", "lor.new", "sd"),
+   model.file = BayesianNMAModel,
+   n.chains = 3, n.iter = 50000, n.burnin = 20000, n.thin = 2,
+   bugs.directory = bugs.dir)
> out.bugs.smry <- out.bugs$summary
> est.bugs <- CI.lower.bugs <- CI.upper.bugs <-
+   PrI.lower.bugs <- PrI.upper.bugs <- matrix(0, 4, 4)
> for(i in 1:4){
+   for(j in 1:4){
+     if(i < j){
+       est.bugs[j, i] <-
+         out.bugs.smry[paste0("lor[", i, ",", j, "]"), "50%"]
+       est.bugs[i, j] <- -est.bugs[j, i]
+       CI.lower.bugs[j, i] <-
+         out.bugs.smry[paste0("lor[", i, ",", j, "]"),"2.5%"]
+       CI.lower.bugs[i, j] <-
+         -out.bugs.smry[paste0("lor[", i, ",", j, "]"), "97.5%"]
+       CI.upper.bugs[j, i] <-
+         out.bugs.smry[paste0("lor[", i, ",", j, "]"), "97.5%"]
+       CI.upper.bugs[i, j] <-
+         -out.bugs.smry[paste0("lor[", i, ",", j, "]"), "2.5%"]
+       PrI.lower.bugs[j, i] <-
+         out.bugs.smry[paste0("lor.new[", i, ",", j, "]"), "2.5%"]
+       PrI.lower.bugs[i, j] <-
+         -out.bugs.smry[paste0("lor.new[", i, ",", j, "]"), "97.5%"]
+       PrI.upper.bugs[j, i] <-
+         out.bugs.smry[paste0("lor.new[", i, ",", j, "]"), "97.5%"]
+       PrI.upper.bugs[i, j] <-
+         -out.bugs.smry[paste0("lor.new[", i, ",", j, "]"), "2.5%"]
+     }
+   }
+ }
```

Here, the first line `set.seed(1234)` specifies a seed to generate random numbers for starting the Markov chain Monte Carlo algorithm, so that all results can be exactly reproduced. The argument `model.file = BayesianNMAModel` in the function `bugs()` specifies the BUGS model; here, recall that `BayesianNMAModel` is an R function containing the BUGS model as defined above. If a separate text file is used to specify the BUGS model, the argument `model.file` should be a character string of the text file's name (and the path to this file if the working directory is not pre-specified). In addition, the argument `bugs.dir` specifies the path to the location of the WinBUGS software program on the user's PC; usually, it has the form of `".../WinBUGS14"`.

The results are obtained using three Markov chains (specified by the argument `n.chains`), each having 30,000 iterations (which is the argument `n.iter` minus the argument `n.burnin`) after a 20,000-

© 2019 Lin L. *JAMA Network Open.*

run burn-in period (specified by the argument `n.burnin`) with thinning rate 2 (specified by the argument `n.thin`) for reducing sample autocorrelations. The results can be displayed as follows:

```
> est.bugs
       [,1]    [,2]    [,3]    [,4]
[1,] 0.0000 -0.4823 -0.8357 -1.0940
[2,] 0.4823  0.0000 -0.3558 -0.6093
[3,] 0.8357  0.3558  0.0000 -0.2541
[4,] 1.0940  0.6093  0.2541  0.0000
> CI.lower.bugs
           [,1]      [,2]       [,3]      [,4]
[1,]  0.0000000 -1.290025 -1.3360000 -1.996000
[2,] -0.2917000  0.000000 -1.1740000 -1.598025
[3,]  0.3954000 -0.451500  0.0000000 -1.083000
[4,]  0.2681825 -0.322000 -0.5437125  0.000000
> CI.upper.bugs
         [,1]     [,2]    [,3]       [,4]
[1,] 0.000000 0.291700 -0.3954 -0.2681825
[2,] 1.290025 0.000000  0.4515  0.3220000
[3,] 1.336000 1.174000  0.0000  0.5437125
[4,] 1.996000 1.598025  1.0830  0.0000000
> PrI.lower.bugs
           [,1]      [,2]      [,3]      [,4]
[1,]  0.0000000 -2.390025 -2.644000 -3.065000
[2,] -1.3750000  0.000000 -2.265025 -2.567025
[3,] -0.8928025 -1.538000  0.000000 -2.169025
[4,] -0.7721050 -1.310025 -1.616025  0.000000
> PrI.upper.bugs
         [,1]     [,2]      [,3]     [,4]
[1,] 0.000000 1.375000 0.8928025 0.772105
[2,] 2.390025 0.000000 1.5380000 1.310025
[3,] 2.644000 2.265025 0.0000000 1.616025
[4,] 3.065000 2.567025 2.1690250 0.000000
```

The interpretation of these results is similar to that of the results produced by the R package “netmeta” in the above section. The objects `CI.lower.bugs` and `CI.upper.bugs` represent the lower and upper bounds of 95% credible intervals (under the Bayesian framework), instead of 95% confidence intervals as in the results produced by the R package “netmeta” (under the frequentist framework).

Remarks

As shown in the above statistical code, prediction intervals can be feasibly produced by all three software programs with a few additional commands. The results of the network meta-analysis of smoking cessation are visualized in the Figure in the main content. The point estimates of odds ratios produced by the three software programs are generally similar for each treatment comparison. The lower and upper bounds of the 95% confidence/credible intervals and the 95% prediction intervals produced by different software programs may have some noticeable differences. These differences may be due to the different model assumptions and specifications used in the different software programs for network meta-analysis.

Research reproducibility

The results produced in this article were based on Stata/SE version 13.0, R version 3.5.3, R package “netmeta” version 1.0-1, R package “R2WinBUGS” version 2.1-2, and WinBUGS version 1.4.3.

© 2019 Lin L. *JAMA Network Open.*

eTable 1. Characteristics of Articles on Network Meta-analyses

**Article**	**Year**	**Journal**	**Statistical method used to perform network meta-analysis**	**Prediction intervals reported (yes/no)**
Phung et al.[11]	2010	*JAMA*	Bayesian RE model	No
Anothaisintawee et al.[12]	2011	*JAMA*	Frequentist RE model	No
Castellucci et al.[13]	2014	*JAMA*	Bayesian FE/RE model	No
Johnston et al.[14]	2014	*JAMA*	Bayesian RE model	No
Bratton et al.[15]	2015	*JAMA*	Frequentist RE model	No
Isayama et al.[16]	2016	*JAMA*	Bayesian RE model	No
Khera et al.[17]	2016	*JAMA*	Bayesian RE model	No
Palmer et al.[18]	2016	*JAMA*	Frequentist RE model	No
Tricco et al.[19]	2017	*JAMA*	Frequentist RE model	No
Gregori et al.[20]	2018	*JAMA*	Bayesian RE model	No
Mitra et al.[21]	2018	*JAMA*	Bayesian RE model	No
Zheng et al.[22]	2018	*JAMA*	Bayesian FE/RE model	No
Cipriani et al.[23]	2011	*Lancet*	Bayesian RE model	No
Palmerini et al.[24]	2012	*Lancet*	Bayesian RE model	No
Leucht et al.[25]	2013	*Lancet*	Bayesian RE model	No
Palmer et al.[26]	2015	*Lancet*	Frequentist RE model	No
Palmerini et al.[27]	2015	*Lancet*	Bayesian RE model	No
Singh et al.[28]	2015	*Lancet*	Bayesian FE/RE model	No
Siontis et al.[29]	2015	*Lancet*	Frequentist RE model	No
Cipriani et al.[30]	2016	*Lancet*	Bayesian RE model	No
da Costa et al.[31]	2017	*Lancet*	Bayesian RE model	No
Jinatongthai et al.[32]	2017	*Lancet*	Frequentist RE model	No
Cipriani et al.[33]	2018	*Lancet*	Bayesian RE model	No
Wandel et al.[34]	2010	*BMJ*	Bayesian RE model	No
Baldwin et al.[35]	2011	*BMJ*	Bayesian/frequentist RE model	No
Hartling et al.[36]	2011	*BMJ*	Bayesian RE model	No
Trelle et al.[37]	2011	*BMJ*	Bayesian RE model	No
Bangalore et al.[38]	2012	*BMJ*	Bayesian RE model	No
Daniels et al.[39]	2012	*BMJ*	Bayesian FE/RE model	No
Haas et al.[40]	2012	*BMJ*	Bayesian RE model	Yes
Hutton et al.[41]	2012	*BMJ*	Bayesian RE model	Yes
Bangalore et al.[42]	2013	*BMJ*	Bayesian RE model	No
Castellucci et al.[43]	2013	*BMJ*	Bayesian RE model	No
Chatterjee et al.[44]	2013	*BMJ*	Bayesian RE model	No[a]
Naci and Ioannidis[45]	2013	*BMJ*	Bayesian RE model	No
Navarese et al.[46]	2013	*BMJ*	Bayesian RE model	No
Stegeman et al.[47]	2013	*BMJ*	Frequentist RE model	No
Uthman et al.[48]	2013	*BMJ*	Bayesian RE model	No
Wu et al.[49]	2013	*BMJ*	Bayesian RE model	No
Bangalore et al.[50]	2014	*BMJ*	Frequentist RE model	No
Loymans et al.[51]	2014	*BMJ*	Bayesian RE model	No
Naci et al.[52]	2014	*BMJ*	Bayesian FE/RE model	No
Price et al.[53]	2014	*BMJ*	Bayesian RE model	No
Tricco et al.[54]	2014	*BMJ*	Frequentist RE model	Yes
Windecker et al.[55]	2014	*BMJ*	Bayesian RE model	No
Alfirevic et al.[56]	2015	*BMJ*	Bayesian FE/RE model	No
Giacoppo et al.[57]	2015	*BMJ*	Bayesian RE model	No
Li et al.[58]	2015	*BMJ*	Bayesian RE model	No

© 2019 Lin L. *JAMA Network Open.*

Article	Year	Journal	Statistical method used to perform network meta-analysis	Prediction intervals reported (yes/no)
Luangasanatip et al.[59]	2015	*BMJ*	Bayesian RE model	No
Dulai et al.[60]	2016	*BMJ*	Bayesian RE model	No
Hazlewood et al.[61]	2016	*BMJ*	Bayesian RE model	No
Lopez-Lopez et al.[62]	2017	*BMJ*	Bayesian FE model	No
Lopez-Lopez et al.[63]	2017	*BMJ*	Bayesian FE model	No
Moser et al.[64]	2017	*BMJ*	Frequentist RE model	No
Wang et al.[65]	2017	*BMJ*	Frequentist RE model	Yes
Siontis et al.[66]	2018	*BMJ*	Frequentist RE model	No
Tricco et al.[67]	2018	*BMJ*	Bayesian RE model	Yes
Xu et al.[68]	2018	*BMJ*	Bayesian RE model	No

[a]Prediction intervals were reported only for pairwise meta-analyses, not for network meta-analyses.
Acronym: FE, fixed effects; NA, not applicable; RE, random effects.
These articles are sorted by their journals, publication years, and first authors' surnames.

© 2019 Lin L. *JAMA Network Open.*

eTable 2. Summary of Software Programs for Producing Prediction Intervals in Network Meta-analysis

Software program	Model type	Command for producing prediction intervals
Stata	Frequentist	Use `intervalplot` with the `predictions` option after running the `network meta` command
R package "netmeta"	Frequentist	Specify the argument `prediction` as `TRUE` in the function `netmeta()`
WinBUGS[a]	Bayesian	Within the BUGS model, draw further samples from the predictive distribution $\delta_{jk,\mathrm{new}} \sim N(d_{jk}, \sigma^2)$ for the treatment comparison of k vs. j, where d_{jk} and σ^2 are the overall relative effect and between-study variance; form the prediction interval based on the posterior quantiles of $\delta_{jk,\mathrm{new}}$

[a]Other software programs that support the Markov chain Monte Carlo algorithm (e.g., JAGS, OpenBUGS, SAS, Stan) can be also used to perform Bayesian network meta-analyses, and thus readily produce prediction intervals.

© 2019 Lin L. *JAMA Network Open.*

eFigure. Estimated Overall Odds Ratios of 6 Treatment Comparisons in the Network Meta-analysis of Smoking Cessation Using Stata, R Package netmeta, and WinBUGS

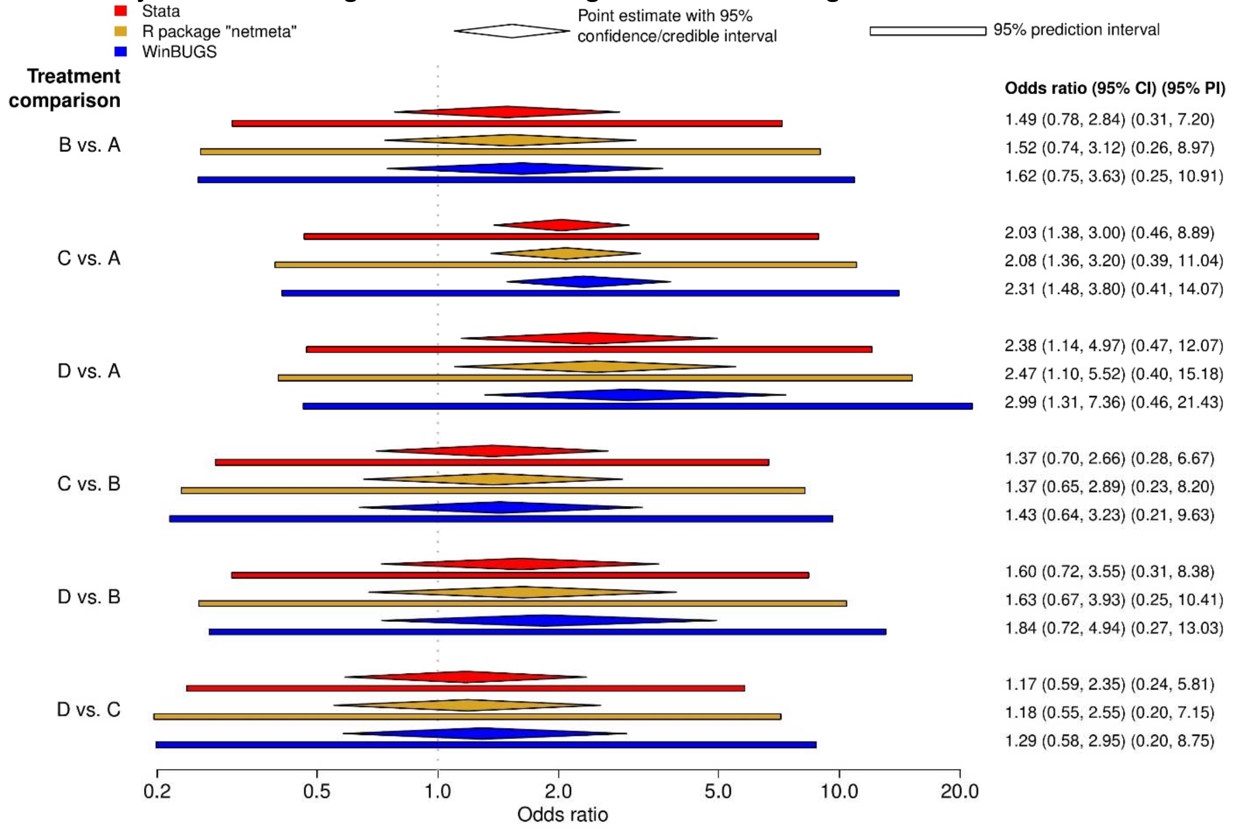

A indicates no intervention; B, self-help; C, individual counseling; and D, group counseling.

© 2019 Lin L. *JAMA Network Open.*

eReferences

1. Nikolakopoulou A, Chaimani A, Veroniki AA, Vasiliadis HS, Schmid CH, Salanti G. Characteristics of networks of interventions: a description of a database of 186 published networks. *PLOS ONE.* 2014;9(1):e86754.
2. Bafeta A, Trinquart L, Seror R, Ravaud P. Analysis of the systematic reviews process in reports of network meta-analyses: methodological systematic review. *BMJ.* 2013;347:f3675.
3. Bafeta A, Trinquart L, Seror R, Ravaud P. Reporting of results from network meta-analyses: methodological systematic review. *BMJ.* 2014;348:g1741.
4. Trinquart L, Attiche N, Bafeta A, Porcher R, Ravaud P. Uncertainty in treatment rankings: reanalysis of network meta-analyses of randomized trialsuncertainty in treatment rankings from network meta-analyses. *Annals of Internal Medicine.* 2016;164(10):666-673.
5. Chaimani A, Higgins JPT, Mavridis D, Spyridonos P, Salanti G. Graphical tools for network meta-analysis in STATA. *PLOS ONE.* 2013;8(10):e76654.
6. Chaimani A, Salanti G. Visualizing assumptions and results in network meta-analysis: the network graphs package. *Stata Journal.* 2015;15(4):905-950.
7. White IR. Network meta-analysis. *Stata Journal.* 2015;15(4):951-985.
8. Dias S, Sutton AJ, Welton NJ, Ades AE. Evidence synthesis for decision making 3: heterogeneity—subgroups, meta-regression, bias, and bias-adjustment. *Medical Decision Making.* 2013;33(5):618-640.
9. Lu G, Ades AE. Combination of direct and indirect evidence in mixed treatment comparisons. *Statistics in Medicine.* 2004;23(20):3105-3124.
10. Dias S, Sutton AJ, Ades AE, Welton NJ. Evidence synthesis for decision making 2: a generalized linear modeling framework for pairwise and network meta-analysis of randomized controlled trials. *Medical Decision Making.* 2013;33(5):607-617.
11. Phung OJ, Scholle JM, Talwar M, Coleman CI. Effect of noninsulin antidiabetic drugs added to metformin therapy on glycemic control, weight gain, and hypoglycemia in type 2 diabetes. *JAMA.* 2010;303(14):1410-1418.
12. Anothaisintawee T, Attia J, Nickel JC, et al. Management of chronic prostatitis/chronic pelvic pain syndrome: a systematic review and network meta-analysis. *JAMA.* 2011;305(1):78-86.
13. Castellucci LA, Cameron C, Le Gal G, et al. Clinical and safety outcomes associated with treatment of acute venous thromboembolism: a systematic review and meta-analysis. *JAMA.* 2014;312(11):1122-1135.
14. Johnston BC, Kanters S, Bandayrel K, et al. Comparison of weight loss among named diet programs in overweight and obese adults: a meta-analysis. *JAMA.* 2014;312(9):923-933.
15. Bratton DJ, Gaisl T, Wons AM, Kohler M. CPAP vs mandibular advancement devices and blood pressure in patients with obstructive sleep apnea: a systematic review and meta-analysis. *JAMA.* 2015;314(21):2280-2293.
16. Isayama T, Iwami H, McDonald S, Beyene J. Association of noninvasive ventilation strategies with mortality and bronchopulmonary dysplasia among preterm infants: a systematic review and meta-analysis. *JAMA.* 2016;316(6):611-624.
17. Khera R, Murad MH, Chandar AK, et al. Association of pharmacological treatments for obesity with weight loss and adverse events: a systematic review and meta-analysis. *JAMA.* 2016;315(22):2424-2434.
18. Palmer SC, Mavridis D, Nicolucci A, et al. Comparison of clinical outcomes and adverse events associated with glucose-lowering drugs in patients with type 2 diabetes: a meta-analysis. *JAMA.* 2016;316(3):313-324.
19. Tricco AC, Thomas SM, Veroniki AA, et al. Comparisons of interventions for preventing falls in older adults: a systematic review and meta-analysis. *JAMA.* 2017;318(17):1687-1699.
20. Gregori D, Giacovelli G, Minto C, et al. Association of pharmacological treatments with long-term pain control in patients with knee osteoarthritis: a systematic review and meta-analysis. *JAMA.* 2018;320(24):2564-2579.
21. Mitra S, Florez ID, Tamayo ME, et al. Association of placebo, indomethacin, ibuprofen, and acetaminophen with closure of hemodynamically significant patent ductus arteriosus in preterm infants: a systematic review and meta-analysis. *JAMA.* 2018;319(12):1221-1238.

© 2019 Lin L. *JAMA Network Open.*

22. Zheng SL, Roddick AJ, Aghar-Jaffar R, et al. Association between use of sodium-glucose cotransporter 2 inhibitors, glucagon-like peptide 1 agonists, and dipeptidyl peptidase 4 inhibitors with all-cause mortality in patients with type 2 diabetes: a systematic review and meta-analysis. *JAMA.* 2018;319(15):1580-1591.
23. Cipriani A, Barbui C, Salanti G, et al. Comparative efficacy and acceptability of antimanic drugs in acute mania: a multiple-treatments meta-analysis. *The Lancet.* 2011;378(9799):1306-1315.
24. Palmerini T, Biondi-Zoccai G, Riva DD, et al. Stent thrombosis with drug-eluting and bare-metal stents: evidence from a comprehensive network meta-analysis. *The Lancet.* 2012;379(9824):1393-1402.
25. Leucht S, Cipriani A, Spineli L, et al. Comparative efficacy and tolerability of 15 antipsychotic drugs in schizophrenia: a multiple-treatments meta-analysis. *The Lancet.* 2013;382(9896):951-962.
26. Palmer SC, Mavridis D, Navarese E, et al. Comparative efficacy and safety of blood pressure-lowering agents in adults with diabetes and kidney disease: a network meta-analysis. *The Lancet.* 2015;385(9982):2047-2056.
27. Palmerini T, Benedetto U, Bacchi-Reggiani L, et al. Mortality in patients treated with extended duration dual antiplatelet therapy after drug-eluting stent implantation: a pairwise and Bayesian network meta-analysis of randomised trials. *The Lancet.* 2015;385(9985):2371-2382.
28. Singh JA, Cameron C, Noorbaloochi S, et al. Risk of serious infection in biological treatment of patients with rheumatoid arthritis: a systematic review and meta-analysis. *The Lancet.* 2015;386(9990):258-265.
29. Siontis GCM, Stefanini GG, Mavridis D, et al. Percutaneous coronary interventional strategies for treatment of in-stent restenosis: a network meta-analysis. *The Lancet.* 2015;386(9994):655-664.
30. Cipriani A, Zhou X, Del Giovane C, et al. Comparative efficacy and tolerability of antidepressants for major depressive disorder in children and adolescents: a network meta-analysis. *The Lancet.* 2016.
31. da Costa BR, Reichenbach S, Keller N, et al. Effectiveness of non-steroidal anti-inflammatory drugs for the treatment of pain in knee and hip osteoarthritis: a network meta-analysis. *The Lancet.* 2017;390(10090):e21-e33.
32. Jinatongthai P, Kongwatcharapong J, Foo CY, et al. Comparative efficacy and safety of reperfusion therapy with fibrinolytic agents in patients with ST-segment elevation myocardial infarction: a systematic review and network meta-analysis. *The Lancet.* 2017;390(10096):747-759.
33. Cipriani A, Furukawa TA, Salanti G, et al. Comparative efficacy and acceptability of 21 antidepressant drugs for the acute treatment of adults with major depressive disorder: a systematic review and network meta-analysis. *The Lancet.* 2018;391(10128):1357-1366.
34. Wandel S, Jüni P, Tendal B, et al. Effects of glucosamine, chondroitin, or placebo in patients with osteoarthritis of hip or knee: network meta-analysis. *BMJ.* 2010;341:c4675.
35. Baldwin D, Woods R, Lawson R, Taylor D. Efficacy of drug treatments for generalised anxiety disorder: systematic review and meta-analysis. *BMJ.* 2011;342:d1199.
36. Hartling L, Fernandes RM, Bialy L, et al. Steroids and bronchodilators for acute bronchiolitis in the first two years of life: systematic review and meta-analysis. *BMJ.* 2011;342:d1714.
37. Trelle S, Reichenbach S, Wandel S, et al. Cardiovascular safety of non-steroidal anti-inflammatory drugs: network meta-analysis. *BMJ.* 2011;342:c7086.
38. Bangalore S, Kumar S, Fusaro M, et al. Outcomes with various drug eluting or bare metal stents in patients with diabetes mellitus: mixed treatment comparison analysis of 22 844 patient years of follow-up from randomised trials. *BMJ.* 2012;345:e5170.
39. Daniels JP, Middleton LJ, Champaneria R, et al. Second generation endometrial ablation techniques for heavy menstrual bleeding: network meta-analysis. *BMJ.* 2012;344:e2564.
40. Haas DM, Caldwell DM, Kirkpatrick P, McIntosh JJ, Welton NJ. Tocolytic therapy for preterm delivery: systematic review and network meta-analysis. *BMJ.* 2012;345:e6226.
41. Hutton B, Joseph L, Fergusson D, Mazer CD, Shapiro S, Tinmouth A. Risks of harms using antifibrinolytics in cardiac surgery: systematic review and network meta-analysis of randomised and observational studies. *BMJ.* 2012;345:e5798.

© 2019 Lin L. *JAMA Network Open.*

42. Bangalore S, Toklu B, Amoroso N, et al. Bare metal stents, durable polymer drug eluting stents, and biodegradable polymer drug eluting stents for coronary artery disease: mixed treatment comparison meta-analysis. *BMJ.* 2013;347:f6625.
43. Castellucci LA, Cameron C, Le Gal G, et al. Efficacy and safety outcomes of oral anticoagulants and antiplatelet drugs in the secondary prevention of venous thromboembolism: systematic review and network meta-analysis. *BMJ.* 2013;347:f5133.
44. Chatterjee S, Biondi-Zoccai G, Abbate A, et al. Benefits of β blockers in patients with heart failure and reduced ejection fraction: network meta-analysis. *BMJ.* 2013;346:f55.
45. Naci H, Ioannidis JPA. Comparative effectiveness of exercise and drug interventions on mortality outcomes: metaepidemiological study. *BMJ.* 2013;347:f5577.
46. Navarese EP, Tandjung K, Claessen B, et al. Safety and efficacy outcomes of first and second generation durable polymer drug eluting stents and biodegradable polymer biolimus eluting stents in clinical practice: comprehensive network meta-analysis. *BMJ.* 2013;347:f6530.
47. Stegeman BH, de Bastos M, Rosendaal FR, et al. Different combined oral contraceptives and the risk of venous thrombosis: systematic review and network meta-analysis. *BMJ.* 2013;347:f5298.
48. Uthman OA, van der Windt DA, Jordan JL, et al. Exercise for lower limb osteoarthritis: systematic review incorporating trial sequential analysis and network meta-analysis. *BMJ.* 2013;347:f5555.
49. Wu H-Y, Huang J-W, Lin H-J, et al. Comparative effectiveness of renin-angiotensin system blockers and other antihypertensive drugs in patients with diabetes: systematic review and bayesian network meta-analysis. *BMJ.* 2013;347:f6008.
50. Bangalore S, Toklu B, Kotwal A, et al. Anticoagulant therapy during primary percutaneous coronary intervention for acute myocardial infarction: a meta-analysis of randomized trials in the era of stents and $P2Y_{12}$ inhibitors. *BMJ.* 2014;349:g6419.
51. Loymans RJB, Gemperli A, Cohen J, et al. Comparative effectiveness of long term drug treatment strategies to prevent asthma exacerbations: network meta-analysis. *BMJ.* 2014;348:g3009.
52. Naci H, Dias S, Ades AE. Industry sponsorship bias in research findings: a network meta-analysis of LDL cholesterol reduction in randomised trials of statins. *BMJ.* 2014;349:g5741.
53. Price R, MacLennan G, Glen J. Selective digestive or oropharyngeal decontamination and topical oropharyngeal chlorhexidine for prevention of death in general intensive care: systematic review and network meta-analysis. *BMJ.* 2014;348:g2197.
54. Tricco AC, Ashoor HM, Antony J, et al. Safety, effectiveness, and cost effectiveness of long acting versus intermediate acting insulin for patients with type 1 diabetes: systematic review and network meta-analysis. *BMJ.* 2014;349:g5459.
55. Windecker S, Stortecky S, Stefanini GG, et al. Revascularisation versus medical treatment in patients with stable coronary artery disease: network meta-analysis. *BMJ.* 2014;348:g3859.
56. Alfirevic Z, Keeney E, Dowswell T, et al. Labour induction with prostaglandins: a systematic review and network meta-analysis. *BMJ.* 2015;350:h217.
57. Giacoppo D, Gargiulo G, Aruta P, Capranzano P, Tamburino C, Capodanno D. Treatment strategies for coronary in-stent restenosis: systematic review and hierarchical Bayesian network meta-analysis of 24 randomised trials and 4880 patients. *BMJ.* 2015;351:h5392.
58. Li B-Z, Threapleton DE, Wang J-Y, et al. Comparative effectiveness and tolerance of treatments for *Helicobacter pylori*: systematic review and network meta-analysis. *BMJ.* 2015;351:h4052.
59. Luangasanatip N, Hongsuwan M, Limmathurotsakul D, et al. Comparative efficacy of interventions to promote hand hygiene in hospital: systematic review and network meta-analysis. *BMJ.* 2015;351:h3728.
60. Dulai PS, Singh S, Marquez E, et al. Chemoprevention of colorectal cancer in individuals with previous colorectal neoplasia: systematic review and network meta-analysis. *BMJ.* 2016;355:i6188.
61. Hazlewood GS, Barnabe C, Tomlinson G, Marshall D, Devoe D, Bombardier C. Methotrexate monotherapy and methotrexate combination therapy with traditional and biologic disease modifying antirheumatic drugs for rheumatoid arthritis: abridged Cochrane systematic review and network meta-analysis. *BMJ.* 2016;353:i1777.
62. López-López JA, Humphriss RL, Beswick AD, et al. Choice of implant combinations in total hip replacement: systematic review and network meta-analysis. *BMJ.* 2017;359:j4651.

© 2019 Lin L. *JAMA Network Open.*

63. López-López JA, Sterne JAC, Thom HHZ, et al. Oral anticoagulants for prevention of stroke in atrial fibrillation: systematic review, network meta-analysis, and cost effectiveness analysis. *BMJ.* 2017;359:j5058.
64. Moser W, Schindler C, Keiser J. Efficacy of recommended drugs against soil transmitted helminths: systematic review and network meta-analysis. *BMJ.* 2017;358:j4307.
65. Wang R, Kim BV, van Wely M, et al. Treatment strategies for women with WHO group II anovulation: systematic review and network meta-analysis. *BMJ.* 2017;356:j138.
66. Siontis GC, Mavridis D, Greenwood JP, et al. Outcomes of non-invasive diagnostic modalities for the detection of coronary artery disease: network meta-analysis of diagnostic randomised controlled trials. *BMJ.* 2018;360:k504.
67. Tricco AC, Zarin W, Cardoso R, et al. Efficacy, effectiveness, and safety of herpes zoster vaccines in adults aged 50 and older: systematic review and network meta-analysis. *BMJ.* 2018;363:k4029.
68. Xu C, Chen Y-P, Du X-J, et al. Comparative safety of immune checkpoint inhibitors in cancer: systematic review and network meta-analysis. *BMJ.* 2018;363:k4226.

© 2019 Lin L. *JAMA Network Open.*
